# Dihydrotestosterone and Finasteride Effects on Alcohol Cue‐Elicited Brain Activity in Males With Heavy Episodic Drinking

**DOI:** 10.1111/adb.70123

**Published:** 2026-01-28

**Authors:** Rafat Boroumand‐Jazi, Sabine Hoffmann, Iris Reinhard, Patrick Bach, Wolfgang H. Sommer, Marlene Kundlacz, Christian P. Müller, Matthias Reichl, Haoye Tan, Leonard P. Wenger, Anne Beck, Sabine Vollstädt‐Klein, Falk Kiefer, Christiane Mühle, Sarah Gerhardt, Bernd Lenz

**Affiliations:** ^1^ Department of Addictive Behavior and Addiction Medicine, Central Institute of Mental Health (CIMH), Medical Faculty Mannheim Heidelberg University Mannheim Germany; ^2^ Biostatistics, Central Institute of Mental Health (CIMH), Medical Faculty Mannheim Heidelberg University Mannheim Germany; ^3^ German Center for Mental Health Partner Site Mannheim‐Heidelberg‐Ulm Germany; ^4^ Institute of Psychopharmacology, Central Institute of Mental Health (CIMH), Medical Faculty Mannheim Heidelberg University Mannheim Germany; ^5^ Bethanien Hospital for Psychiatry, Psychotherapy and Psychosomatic Medicine Greifswald Germany; ^6^ Department of Psychiatry and Psychotherapy Friedrich‐Alexander‐Universität Erlangen‐Nürnberg and Universitätsklinikum Erlangen Erlangen Germany; ^7^ Institute for Mental Health and Behavioral Medicine, Department of Psychology HMU Health and Medical University Potsdam Potsdam Germany; ^8^ Mannheim Center for Translational Neurosciences (MCTN), Medical Faculty Mannheim Heidelberg University Mannheim Germany

**Keywords:** alcohol use disorder, androgens, cue reactivity, dihydrotestosterone, finasteride, fMRI

## Abstract

Preliminary animal and human studies have shown that blood dihydrotestosterone concentrations are increased in males with alcohol use disorder, and 5α‐reductase inhibitors, which decrease dihydrotestosterone concentrations, reduce alcohol consumption. To gain mechanistic insight, we studied the effects of reduced dihydrotestosterone concentrations following pharmacological 5α‐reductase inhibition on alcohol cue‐elicited brain activity and alcohol craving in males with problematic alcohol use. To this end, this randomized, placebo‐controlled, crossover challenge experiment investigated associations between dihydrotestosterone concentrations and brain functional magnetic resonance imaging (fMRI) activity during exposure to visual alcohol cues and alcohol craving following a single dose of 5 mg finasteride versus placebo in 50 males with heavy episodic drinking. We used finasteride because it specifically inhibits 5α‐reductase II activity, which is the main enzyme converting testosterone to dihydrotestosterone. Dihydrotestosterone concentrations were lower in the finasteride condition in comparison to the placebo condition, but not significantly associated with brain activation patterns or craving. In the exploratory analyses, we found higher brain activity during exposure to visual stimuli in the right and left caudate nuclei, the right superior frontal gyrus and the left insula in the finasteride condition versus the placebo condition. Moreover, finasteride versus placebo was associated with a higher wish to not drink alcohol. The results of this experimental study do not support the à priori hypothesis that dihydrotestosterone concentrations play a role in brain activation during exposure to visual alcohol cues, but indicate that finasteride effects may be mediated by other pathways. Future studies are requested to investigate the effects of reduced dihydrotestosterone concentrations over a longer time and to shed light on the molecular mechanisms underlying the here observed effects of finasteride.

**Trial Registration:** DRKS00020569

## Graphical abstract

In a randomized, double‐blind, placebo‐controlled crossover study, we investigated whether finasteride‐induced reduction of dihydrotestosterone alters alcohol cue‐elicited brain activity and craving in males with heavy episodic drinking. Although dihydrotestosterone concentrations were not associated with neural cue reactivity, finasteride increased brain activation in the right and left caudate nuclei, right superior frontal gyrus, and left insula in response to visual alcohol cues, and enhanced the wish to not drink alcohol. These findings suggest that finasteride effects on alcohol cue processing may be mediated by dihydrotestosterone‐independent neurobiological mechanisms.

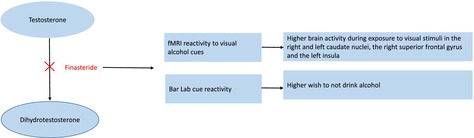

## 1 Introduction

Alcohol consumption is a significant global health problem that results in a high somatic and psychological disease burden. The 2015 Addiction Survey revealed that worldwide 22.4% of men aged 18–64 years consume alcohol at a risky level and 46.5% engage in heavy episodic drinking [1].

Dihydrotestosterone blood concentrations were found to be higher in early‐abstinent alcohol‐dependent male patients in comparison to healthy male controls, and an increase in dihydrotestosterone concentrations during the first 5 days of abstinence predicted more alcohol‐related hospital readmissions in the following 12 months [2]. These preliminary results suggest a role of dihydrotestosterone concentrations in alcohol use disorder. However, there is a need for a better understanding of the underlying neurobiological mechanisms.

The 5α‐reductase enzyme catalyses the conversion of testosterone to its metabolite dihydrotestosterone with an increased affinity to the androgen receptor [3, 4]. Animal and preliminary human studies indicate that the 5α‐reductase inhibitors finasteride and dutasteride might protect against problematic alcohol use. Rodent experiments show that finasteride decreases alcohol intake, prevents the development of preference to alcohol and inhibits the acquisition of alcohol consumption in a dose‐dependent manner in male mice [5, 6]. Also, dutasteride was found to attenuate the sedating effect of alcohol and to reduce total as well as heavy alcohol use in heavy drinkers [7]. These effects of 5α‐reductase inhibitors may be mediated by attenuating dihydrotestosterone concentrations. However, 5α‐reductase inhibitors can also influence neurosteroid levels of allopregnanolone [8]. The effects of 5α‐reductase inhibitors may therefore be mediated not only by attenuating dihydrotestosterone concentrations but also by altering levels of allopregnanolone.

Neuroanatomical explanatory models for high alcohol consumption focus mostly on prefrontal brain regions and the mesolimbic reward system [9], although many other brain areas were shown to be also involved in alcohol use disorder [10]. In general, exposure to visual alcohol cues in patients with alcohol use disorder modulates activation in the left middle occipital gyrus and cerebellum, left inferior frontal gyrus and insula, right middle occipital gyrus, right angular gyrus and superior parietal gyrus, left supplementary motor area, right posterior cingulate gyrus, right precuneus, right middle temporal gyrus and right superior parietal gyrus (see table 2, [10]). These neural circuitries influence behavioural control as well as the experience of reward, motivation and reinforcement in response to alcohol cue exposure. Alcohol stimuli play a significant role in the development and maintenance of alcohol use disorder and have a direct effect on the mesolimbic reward system in the brain. A recent meta‐analysis identified the following brain regions to exhibit significant changes after undergoing AUD treatment (psychosocial and pharmacological intervention) [10]. As compared to placebo treatment or when comparing pre to post intervention, increased activation in the right precentral gyrus and decreased activations in the right middle frontal gyrus, bilateral caudate nucleus, bilateral insula and bilateral superior frontal gyrus were reported.

### 1.1 Study Aims

Initial evidence suggests a role of dihydrotestosterone in alcohol use disorder. However, there is a research gap regarding the underlying mechanisms. The primary aim of this pre‐registered, randomized, placebo‐controlled, double‐blind, crossover finasteride pharmacological challenge experiment in males with heavy alcohol drinking was to investigate whether the reduction in dihydrotestosterone concentrations following finasteride in a dose of 5 mg (versus placebo) affects brain activation during exposure to visual alcohol stimuli and alcohol craving. We hypothesized that the reduction in dihydrotestosterone concentrations associates with changes in activation to visual alcohol cues in the brain areas identified by the aforementioned meta‐analysis [10] as well as in craving. As positive and negative controls, we studied finasteride‐induced changes in androgens and allopregnanolone and expected decreased dihydrotestosterone concentrations [11] without significant changes in testosterone and allopregnanolone [12].

## 2 Methods and Materials

### 2.1 Sample

The randomized, placebo‐controlled, double‐blind, crossover experiment was conducted within the framework of the TRR 265 [13, 14]. It was preregistered in the German Trial Registry (identifier: DRKS00020569) and approved by the local ethics committee of the Medical Faculty Mannheim, Heidelberg University (ID: 2021‐654). All participants provided written informed consent. The recruitment took place from February 2022 to February 2023 (first to last study visit). The participants were required to be male and aged between 18 and 65 years, to have a body mass index (BMI) ranging from 18.5 to 30 kg/m^2^, to possess the ability to provide informed consent after detailed information, to have sufficient knowledge of the German language, a breath alcohol concentration of 0.0‰ and to be capable of undergoing functional magnetic resonance imaging (fMRI). They were also required to meet the criteria for heavy episodic drinking as defined by the World Health Organization: a minimum consumption of five alcoholic beverages, each containing 12 g of alcohol (12 g of alcohol is roughly equivalent to 0.33 L of beer or 0.125 L of wine), on a single occasion (i.e., within a few hours), once per month. This was assessed via clinical interview. Exclusion criteria were as follows: lack of written informed consent; medical history with outpatient or inpatient treatment of depressive or bipolar disorder, psychotic disorder, schizophrenia or schizophrenic spectrum disorder, or substance dependence other than alcohol, nicotine or cannabis; medical history of severe head injury or other severe central nervous system disease (e.g., dementia, Parkinson's disease, multiple sclerosis); use of drugs or medications that interact with the central nervous system within the past 10 days or if their use was less than 5 half‐lives ago; regular use of finasteride for medical reasons; exclusion criteria for finasteride use: known hypersensitivity to finasteride, clinically significant pharmacokinetic interaction (finasteride is metabolized via the cytochrome P450‐3A4 system), currently planning pregnancy with sexual intercourse without contraception, pregnant partner; exclusion criteria for an MRI examination (e.g., metal in the body).

### 2.2 Study Design

**FIGURE 1:**
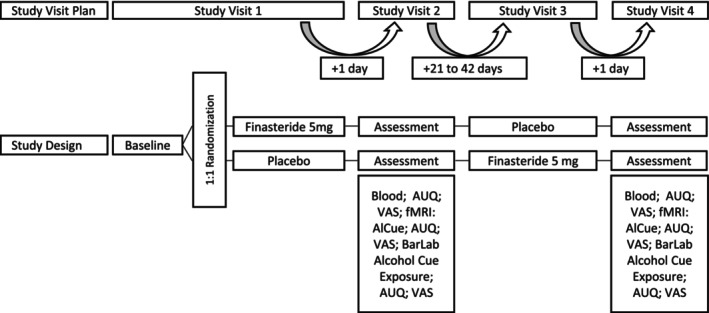
Study design of the randomized, placebo‐controlled, double‐blind, crossover challenge experiment. The participants attended the following assessments during study visits 2 and 4: the Alcohol Urge Questionnaire (AUQ), visual analogue scales (VAS), fMRI Alcohol Cue Exposure Task, bar lab exposure. FIN, finasteride; PLA, placebo.

The experimental design consisted of two blocks, each comprising two study visits, resulting in four visits per participant (V1, V2, V3 and V4). During V1 and V3, either 5 mg finasteride or placebo was administered; the capsule of placebo consisted of 99.5 parts mannitol and 0.5 parts colloidal silicon dioxide (aerosol). fMRI measurements and bar lab alcohol cue exposures took place during V2 and V4 (for details, see Figure 1). Breath alcohol was assessed at the beginning of each study visit, and 0‰ breath alcohol was a precondition for continuing the visit. A standard urine drug screening except for marijuana and tetrahydrocannabinol was conducted during study visits 2 and 4, and a negative drug screening was a precondition to proceed. V1 and V2 as well as V3 and V4 were conducted on consecutive days. There was a wash‐out phase of 21–42 days between V2 and V3 to exclude bias from carry‐over effects, as previous work showed that a single dose of 5 mg finasteride suppresses dihydrotestosterone concentrations in men for up to 2 weeks [11].

### 2.3 fMRI Alcohol Cue Exposure Task (AlCue)

Sixty‐five photos associated with alcohol (encompassing beer, wine, liquor) were introduced together with 40 neutral stimuli, employing a blocked experimental design. The presentation entailed 13 blocks of alcohol‐related stimuli and 8 blocks of neutral stimuli in a pseudo‐randomized order. Each block comprised five individual stimuli, each of which was displayed for a duration of 4 s. This protocol yielded a cumulative temporal extent of 20 s per block. Including the preparatory phase, the duration the task spanned was 12 min and 18 s. The arrangement of photos within the categories ‘alcohol’ and ‘neutral’ was pseudorandomized [15].

Brain scans were conducted at the Central Institute of Mental Health in Mannheim using a 3T whole‐body tomograph (MAGNETOM 3.0T XR Numaris, Siemens Medical Systems, Erlangen, Germany). To obtain comprehensive images of the entire brain, a T2*‐weighted multi‐band echo‐planar imaging (mb‐EPI) sequence with an acceleration factor of 6 was employed. The scans were acquired in a transversal orientation rotated 20° clockwise to the anterior commissure–posterior commissure (AC‐PC) line. Specific imaging parameters were utilized to measure the neural task‐related blood oxygen level‐dependent (BOLD) response: repetition time (TR) 869 ms, echo time (TE) 38 ms, flip angle 58°, 60 slices with a thickness of 2.4 mm, voxel dimensions 2.4 × 2.4 × 2.4 mm^3^, no inter‐slice gap, field of view (FOV) 210 × 210 mm^2^, matrix size 88 × 88, acquisition orientation transversal to coronal (T > C), interleaved slice order, acceleration factor slice 6, a flip angle of 58°, bandwidth 1832 Hz/Px, prescan normalization, weak raw data filtering, LeakBlock kernel and fat saturation. Overall, 840 images were acquired for each participant during the assessment. fMRI data were preprocessed in SPM12 (Statistical Parametrical Mapping; Wellcome Centre for Human Neuroimaging, at University College, London, UK) using a standardized pipeline including slice timing, spatial realignment, normalization (template: SPM12 tissue probability map according to the Montreal Neurological Institute) and spatial smoothing (Gaussian kernel of 8 mm full width at half maximum). Quality checks were performed and individuals with excessive head movement or other artefacts were excluded from further analyses.

### 2.4 Bar Lab

Alcohol cue exposure in a bar lab is an established experimental methodology to investigate how exposure to alcohol‐related cues affects behaviour, particularly craving, in individuals who consume alcohol. In the present study, each participant attended one standardized bar lab session following the fMRI scans at both visits (V2 and V4). During these sessions, participants were alone in a realistic bar‐like environment, under continuous observation via a camera. They were exposed to multiple alcohol‐related cues, including the visual and olfactory presence of their favourite alcoholic beverages. Alcohol‐related objects such as bottles, glasses and bar utensils were also present in the room to enhance cue salience. The exposure period lasted approximately 10–15 min. Importantly, no alcohol consumption occurred during these sessions, and there was no neutral or water control condition. Craving was assessed immediately after the bar lab exposure. For an overview of the assessment timeline, including baseline, post‐MRI and post‐bar lab assessments, see Figure 1.

### 2.5 Behavioural Assessments

The Alcohol Use Disorder Identification Test (AUDIT) assesses alcohol consumption, drinking behaviours and alcohol‐related problems through a series of questions. It measures alcohol use disorder and at‐risk consumption (intraclass correlation coefficient of 0.95 and retest reliability of 0.86), with a sensitivity of 0.97 and specificity of 0.91 in a German representative sample [16].

Behavioural assessments conducted during the study included various measures. To assess alcohol craving among participants, the Alcohol Urge Questionnaire (AUQ) and a series of visual analogue scales (VAS) were employed, designed to capture various dimensions of craving and intention towards alcohol consumption at baseline, after fMRI and after bar lab exposure [17]. The AUQ is a validated tool used to measure the intensity and frequency of alcohol craving (internal consistency 0.91) [18]. It consists of eight items rated on a 7‐point Likert scale, with questions focusing on the participant's desire to drink, expectations of positive effects from drinking and the difficulty in resisting the urge to drink. Additionally, we used VAS as follows: VAS 1 assesses the immediate craving intensity for alcohol, quantifying the urge to drink at the moment. VAS 2 evaluates the participant's current intention to consume alcohol, focusing on decision‐making regarding drinking. VAS 3 examines expectations about the positive effects of alcohol consumption at the moment, that is, mood improvement or social benefits. VAS 4 measures expectations of alcohol's effects on alleviating negative states or withdrawal symptoms and VAS 5 the motivation to abstain from alcohol, shedding light on the participant's desire for sobriety and resistance to craving.

### 2.6 Blood Measures

Blood samples were collected approximately 30 min before each study visit and dihydrotestosterone, testosterone and allopregnanolone concentrations were quantified in serum aliquots stored at −80°C. We determined sex hormone concentrations using the following competitive enzyme‐linked immunosorbent assays (ELISAs) with the indicated serum sample volumes in duplicates, standard ranges and achieved coefficients of variation (cv): 5a‐Dihydrotestosterone ELISA (DB52021; IBL International GmbH, Hamburg, Germany; 50 μL, standard 6–2500 pg/mL, 1.5%, 2.2%); Testosterone ELISA (DRG‐EIA‐1559; DRG Instruments GmbH, Marburg, Germany; 25 μL, standard 0.1–16 ng/mL, 1.9%, 3.4%); and Allopregnanolone AssayMax ELISA Kit (EA1701‐1; AssayMax via biotrend, St. Charles, United States; 5 μL, standard 0.3–640 pg/mL, intra‐assay cv 7.5%, inter‐assay cv 11.2%). Peak suppression of dihydrotestosterone occurs approximately 8 h after finasteride intake and persists for several days. In our study, V2 and V4 were conducted directly on the day following V1 and V3 ensuring that blood sampling occurred during the period of sustained dihydrotestosterone suppression.

### 2.7 Statistical Analyses

#### 2.7.1 Neuroimaging

In a first step, we used general linear mixed models to study the association between dihydrotestosterone concentration (predictor) and brain activation (dependent variable) in response to ‘alcohol’ and ‘neutral’ cues within regions of interest (ROI). Alcohol and neutral cue conditions were modelled separately rather than combined into an ‘alcohol versus neutral’ difference contrast, because previous work showed that alcohol and neutral cues alone exhibit moderate to good test–retest reliability (ICC > 0.40), whereas the ‘alcohol versus neutral’ contrast shows poor overall reliability [19]. Subsequently, we adjusted these models for time point, medication and sequence. As ROIs we defined the brain regions previously identified by a meta‐analysis of treatment effects in AUD [10] (right precentral gyrus, right middle frontal gyrus, bilateral caudate nuclei, bilateral insula and bilateral superior frontal gyri). We exported activities based on calculated sphere sizes. The reported number of voxels for each brain area were transformed into mm^3^ based on the assumption that 1 voxel corresponds to 6.67 mm * 6.67 mm * 6.67 mm (full‐width at half‐maximum [FWHM] = 20 mm) in the meta‐analysis [10], and the radius within the sphere was calculated. To account for the nesting structure of each data point within a participant (Level 1: VAS and blood hormone concentrations; Level 2: participants), the models included random intercepts. The data were analysed using SPSS 27.0 for Windows and visualized using GraphPad Prism 5. *p* < 0.05 (two‐sided) was considered significant.

In a second step, we performed a whole‐brain full factorial analysis in SPM12, incorporating dihydrotestosterone concentration, time point, medication and sequence. The dihydrotestosterone concentration was included as a covariate of interest, with contrasts specified accordingly. Full factorial analyses were conducted to assess the impact of dihydrotestosterone concentrations (quantified in blood from V2 and V4) on brain activity across two conditions: ‘alcohol’ and ‘neutral’. Consistent with the reliability considerations described above, these conditions were entered as separate regressors rather than as an ‘alcohol versus neutral’ contrast. To address the issue of multiple comparisons, we applied a family‐wise error (FWE)‐corrected threshold of *p* < 0.001, which was increased in steps of 0.001, in combination with a cluster‐extent threshold, following the random field theory as implemented in SPM12. During analysis, whole brain data were visualized using MRIcro, a software for magnetic resonance image conversion, viewing and analysis [20].

#### 2.7.2 Behavioural Data

We used general linear mixed models to test associations between dihydrotestosterone concentration (predictor) and AUQ score as well as the five VAS measures (dependent variables). Similar to the neuroimaging approach, we then adjusted these models for time point, medication and sequence.

#### 2.7.3 Positive and Negative Controls

For validation that finasteride versus placebo reduces dihydrotestosterone concentrations, without significant effects on testosterone or allopregnanolone concentrations, we used general linear mixed models with medication, V1/V3 versus V2/V4, medication‐by‐V1/V3 versus V2/V4, time point and time of blood collection as predictors.

## 3 Results

### 3.1 Sample Characteristics

In total, 195 non‐treatment seeking male individuals were assessed for eligibility, 123 individuals were pre‐screened during a brief telephone interview for inclusion and exclusion criteria. Afterwards, 85 eligible participants received a more in‐depth screening, and 50 male individuals were included. All available datasets were used (N[observations] fMRI 84 and craving 282). For further elaboration on available data and dropouts, refer to Figure S1.

The mean age was 34.48 years (SD = 14.00). The AUDIT assessment yielded a mean score of 11.17 (SD = 4.97), indicative of a prevailing pattern of hazardous alcohol consumption within the study cohort. The mean BMI was 24.57 kg/m^2^ (SD = 2.68). A significant majority reported having achieved a high school diploma (69%) and had been in employment within the preceding 3 months (93%, *n* = 46). Of the group, 56% were categorized as single (*n* = 28), 34% were either married or in a committed relationship (*n* = 17), and 4% were either divorced or separated (*n* = 2). Over one‐third of the participants indicated having children (38%, *n* = 19), and slightly more than one‐third were identified as smokers (34%, *n* = 17). The finasteride–placebo and the placebo–finasteride sequences did not significantly differ in sample characteristics, which confirms successful randomization (Table S1).

### 3.2 Region of Interest Activity During Exposure to Visual Alcohol Stimuli

Dihydrotestosterone concentrations were not significantly associated with activation during exposure to visual alcohol stimuli of any of the ROI (Table 1). There was also no significant association between dihydrotestosterone concentrations and brain activation to neutral cues (Table S2).

**TABLE 1:** Association between dihydrotestosterone concentrations and brain activation in separated models specific to the regions of interest indicated.

	*β*	95% CI	*F*	df1; df2	*p*
N[cases] = 48 N[observations] = 84					
Right caudate					
Dihydrotestosterone concentrations	0.000	[−0.002; 0.001]	0.387	1; 57	0.536
Age	−0.005	[−0.014; 0.004]	1.256	1; 47	0.268
Smoking status	−0.149	[−0.384; 0.085]	1.642	1; 45	0.207
Left caudate					
Dihydrotestosterone concentrations	0.000	[−0.001; 0.001]	0.591	1; 56	0.445
Age	−0.008	[−0.015; −0.001]	4.852	1; 45	0.103
Smoking status	−0.155	[−0.347; 0.038]	2.620	1; 43	0.113
Left superior frontal gyrus orbital					
Dihydrotestosterone concentrations	0.000	[−0.002; 0.002]	0.191	1; 65	0.664
Age	−0.005	[−0.019; 0.010]	0.412	1; 49	0.524
Smoking status	−0.335	[−0.711; 0.041]	3.210	1; 46	0.080
Right superior frontal gyrus					
Dihydrotestosterone concentrations	−0.001	[−0.003; 0.000]	3.072	1; 67	0.084
Age	−0.003	[−0.016; 0.010]	0.216	1; 49	0.644
Smoking status	−0.183	[−0.516; 0.149]	1.232	1; 46	0.273
Right middle frontal gyrus					
Dihydrotestosterone concentrations	−0.001	[−0.003; 0.000]	2.259	1; 66	0.138
Age	−0.003	[0.605; −0.015]	0.271	1; 48	0.605
Smoking status	−0.175	[0.265; −0.486]	1.276	1; 46	0.265
Right precentral gyrus					
Dihydrotestosterone concentrations	0.001	[−0.001; 0.003]	0.970	1; 67	0.328
Age	0.000	[−0.014; 0.015]	0.003	1; 46	0.955
Smoking status	−0.119	[−0.501; 0.263]	0.394	1; 43	0.534
Right insula					
Dihydrotestosterone concentrations	−0.001	[−0.002; 0.000]	2.355	1; 63	0.130
Age	−0.002	[−0.011; 0.008]	0.146	1, 47	0.704
Smoking status	−0.317	[−0.569; −0.065]	6.402	1; 45	0.115
Left insula					
Dihydrotestosterone concentrations	0.000	[−0.001; 0.001]	0.046	1; 58	0.832
Age	−0.007	[−0.016; 0.003]	2.062	1; 46	0.158
Smoking status	−0.194	[−0.436; 0.048]	2.599	1; 44	0.114

*Note:* The multilevel models include fixed and random intercepts. 95% CI, 95% confidence interval; β, regression coefficient.

**FIGURE 2:**
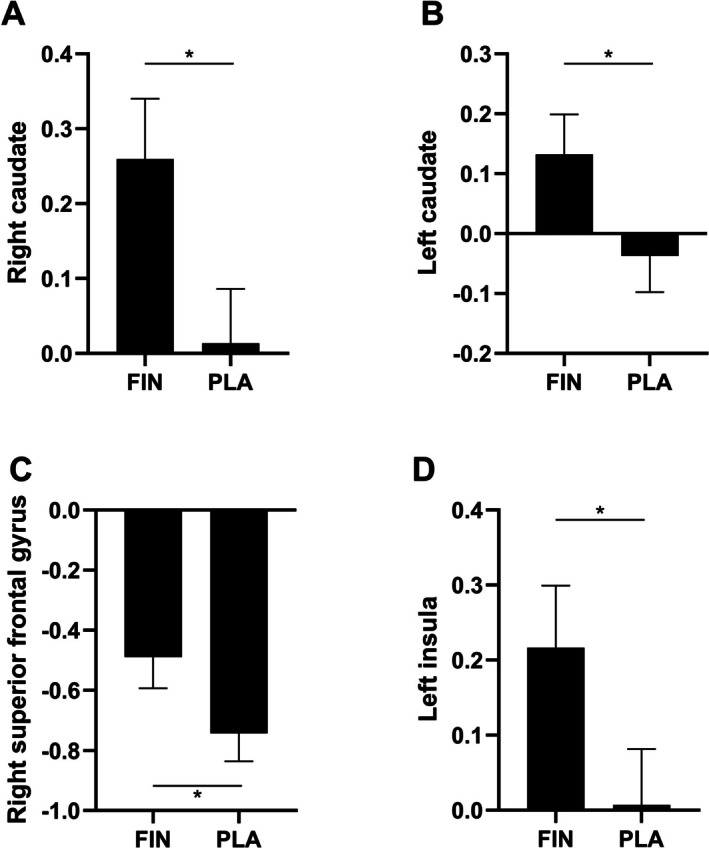
Brain activity during visual alcohol stimuli exposure in the finasteride (FIN) and placebo (PLA) conditions. Higher brain activity was observed in the right and left caudate nuclei, right superior frontal gyrus and left insula in the FIN condition compared to PLA (*p < 0.05).

Also, in models adjusted for time point, medication and sequence, dihydrotestosterone concentrations were not significantly related to brain activation during exposure to visual alcohol stimuli in the specific brain regions. In exploratory analyses, we found higher brain activity during exposure to visual alcohol stimuli in the right and left caudate nuclei, the right superior frontal gyrus and the left insula in the finasteride condition versus the placebo condition (Figure 2 and Table S3). None of the factors was significantly associated with brain activation to neutral cues (Table S4).

### 3.3 Whole Brain Activity During Exposure to Visual Alcohol Stimuli

In the models adjusted for time point, medication and sequence, dihydrotestosterone concentrations were not significantly related to brain activation to alcohol and neutral stimuli. Also, medication was not significantly associated with whole brain activation to alcohol stimuli at a *p* < 0.001.

### 3.4 Alcohol Craving

**FIGURE 3:**
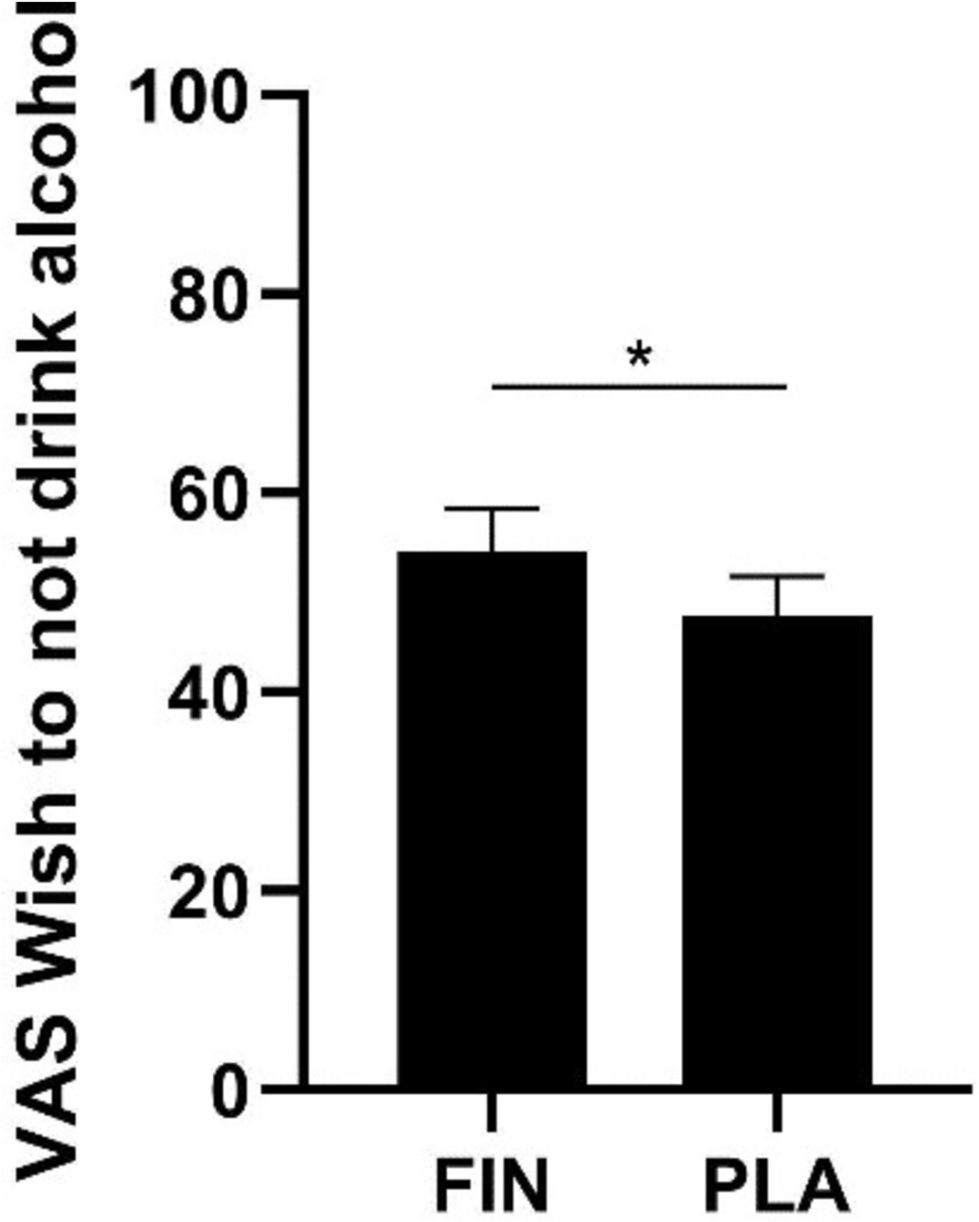
The visual analogue scale (VAS) value for ‘Wish to not drink alcohol’ was higher in the finasteride than in the placebo condition. *p < 0.05.

No significant relationship was found between dihydrotestosterone concentrations and the AUQ scores or VAS scores. Similarly, medication showed no significant association with AUQ or most VAS scores, except for VAS 5, where finasteride demonstrated a significant effect to increase the wish to not drink alcohol. (Figure 3 and Tables S5 and S6).

### 3.5 Positive and Negative Control: Medication Effects on Hormone Parameters

**FIGURE 4:**
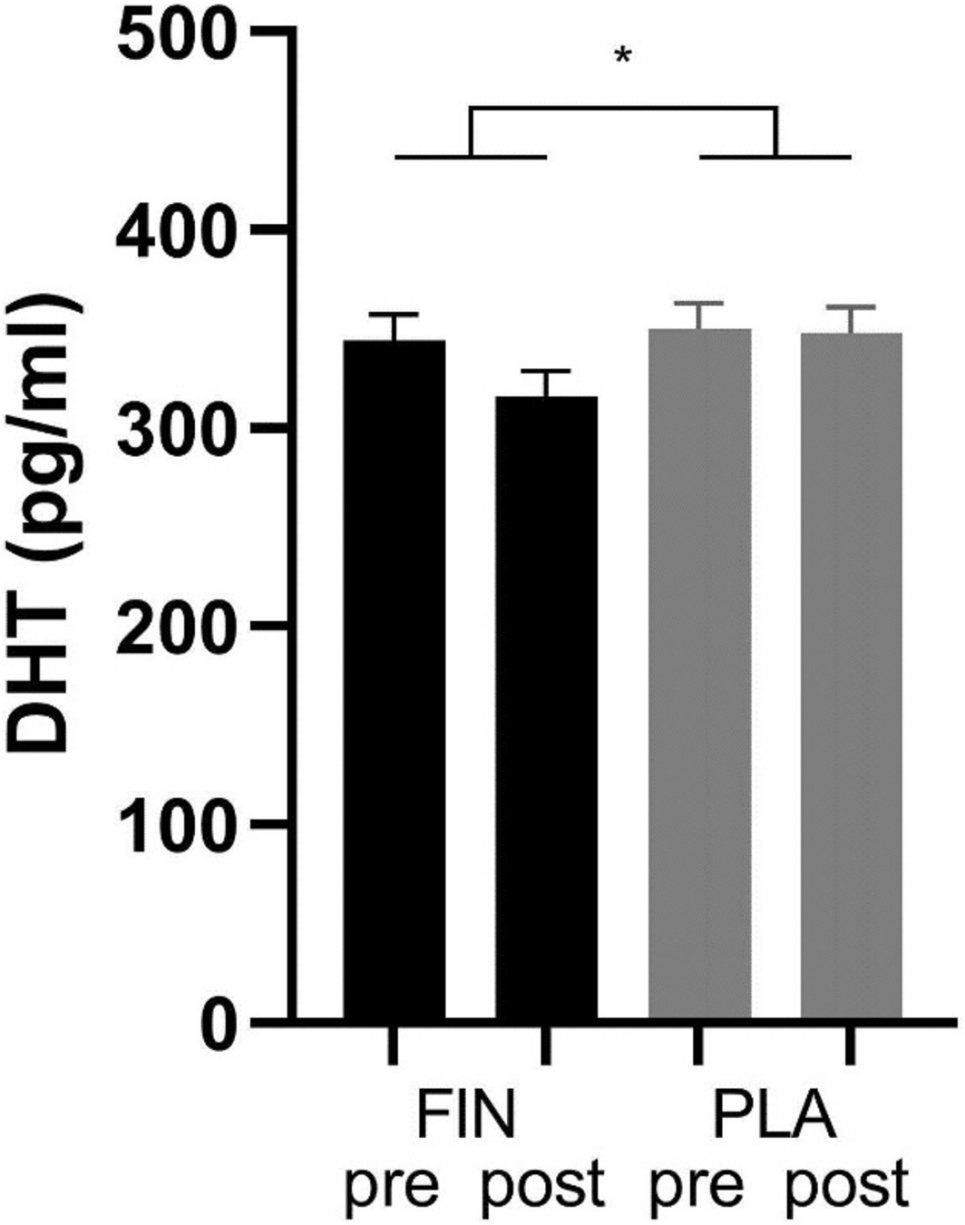
The graph illustrates the moderating effect of finasteride (FIN) versus placebo (PLA) on the difference from study visits 1 (V1) and 3 (V3) (before medication = pre) to study visits 2 (V2) and 4 (V4) (after medication = post) in dihydrotestosterone (DHT) concentrations.

Medication (finasteride versus placebo) significantly moderated the change in dihydrotestosterone concentration from administration of medication (V1 and V3) to the day afterwards (V2 and V4), with a larger decline in the finasteride condition (Figure 4). As expected, there was no such a significant effect for testosterone or allopregnanolone concentrations (Table S7).

## 4 Discussion

In this randomized, placebo‐controlled, double‐blind, crossover finasteride challenge experiment, we addressed the potential association between dihydrotestosterone and its reduction through finasteride with brain activation and alcohol craving. To this end, we investigated the effects of reduced dihydrotestosterone concentrations following a single dose of 5 mg finasteride, an inhibitor of the 5α‐reductase II enzyme, on brain activation during exposure to visual alcohol cues and alcohol craving.

Dihydrotestosterone concentrations were not significantly associated with brain activation patterns during exposure to visual alcohol stimuli or with self‐reported alcohol craving. However, the region of interest analyses indicated that finasteride (versus placebo) was linked to a higher activation in the bilateral caudate nuclei, right superior frontal gyrus and left insula during exposure to alcohol cues.

These regions are critically involved in reward processing, behavioural control and interoception [21]. The exploratory analyses suggest that finasteride may influence neural correlates associated with behavioural control and reward, although our data indicate that this effect is not due to variation in dihydrotestosterone concentrations. Various neurotransmitter systems, such as dopamine and GABA [22], neuroinflammation, hypothalamic–pituitary–adrenal axis activity and epigenetic mechanisms [22, 23] have been shown to be influenced by finasteride. A line of animal experiments has shown that 5α‐reductase inhibitors exert anti‐dopaminergic effects. For example, finasteride was shown to differentially modulate the effects of dopamine D1‐ and D2‐like receptor agonists on behavioural control and sensorimotor gating [24, 25]. Moreover, finasteride was shown to reduce GABA transaminase protein levels in brain tissue of male rats [26]. Also, preclinical and clinical data suggest that 5‐alpha reductase inhibitors may be associated with reduced hippocampal neurogenesis, increased neuroinflammation, altered activity of the HPA axis and epigenetic modifications [22].

The observed effects of finasteride may result from a combination of neurobiological mechanisms independent of dihydrotestosterone. This indicates that finasteride may be a more general neuromodulatory agent. Further exploration of these multifaceted effects is needed to enhance our understanding of how responses to alcohol cues can be influenced by finasteride.

Similarly to the brain effect, dihydrotestosterone concentrations were not related to the alcohol craving measures. However, there was an association of finasteride (versus placebo) with an increased wish to not drink alcohol (VAS 5). This observation suggests that finasteride may enhance cognitive or emotional resistance to alcohol through mechanisms unrelated to dihydrotestosterone. The findings stimulate further research to elucidate the precise mechanisms underlying these effects and their implications for problem drinking.

The findings are important, as alcohol cue reactivity is a relevant factor in the development and persistence of alcohol use disorder [27]. The here presented cue reactivity paradigm is one of the most commonly used ones in functional imaging studies of substance use disorders [28]. Brain responses to drug‐related stimuli are even considered potential biomarkers for diagnostic and prognostic purposes as well as for predicting treatment outcomes [29]. It has been suggested that enhanced striatal cue reactivity might represent a kind of a ‘warning signal’ that may help patients to abstain from further alcohol consumption [30]. Moreover, a higher activation of frontal and striatal brain areas is expected to co‐occur with higher behavioural control [31] for which our VAS item of a higher wish to not drink alcohol might be a proxy.

Consistent with this, Schad and colleagues observed inhibition of approach behaviour by alcohol‐related background stimuli in detoxified alcohol‐dependent patients, which was associated with an increased neural cue response. Interestingly, both effects only occurred in later abstainers, but not in relapsers and individuals with mild but not severe dependence [32].

As a positive control, the reduction in dihydrotestosterone concentrations following the administration of finasteride is consistent with the expected pharmacological action—although a previous study indicates a stronger effect than detected here [33]. Also, as expected, there were no significant changes in testosterone or allopregnanolone concentrations (negative control).

A strength of this project is the randomized, double‐blind, placebo‐controlled, crossover study design. However, only relatively young male subjects with heavy episodic drinking were included. Our hypotheses were built on a previous study of early‐abstinent in‐patients with alcohol dependence undergoing withdrawal treatment [2]. By contrast, the here investigated subjects were non‐treatment seeking individuals with heavy episodic drinking which might explain the null findings. The neural mechanisms of dihydrotestosterone may depend on the severity of AUD and hence not be present in our sample. Therefore, additional studies are needed to investigate the neural and behavioural effects of dihydrotestosterone in males and females with severe alcohol use disorder and other age groups. Also, the rather small sample size requests caution in interpreting the results. Moreover, the craving data is based on self‐reports, which might have induced bias due to social desirability. The significant findings of this study should be interpreted with caution as we conducted a lot of statistical tests and did not correct for multiple hypothesis testing, which might have resulted in false positive findings. Future studies should use psychophysiological measures of craving. The scope of neurosteroid profiling was limited, as measures such as THP, THDOC and pregnenolone sulphate were not assessed; however, these compounds may play an important role in mediating finasteride's neuromodulatory effects. Although this study does not provide evidence for a role of DHT in heavy episodic drinking, it offers important groundwork on the neurobiological effects of finasteride and emphasizes the need for careful population selection in future mechanistic studies targeting individuals with severe AUD.

## 5 Conclusion

Our study highlights the intricate interplay of hormonal, neural and behavioural factors in heavy episodic drinking. The null findings might be a consequence of the specific sample characteristics of heavy drinkers with AUDIT scores indicating mild‐to‐moderate AUD. As dihydrotestosterone concentrations were not correlated with brain activity or craving, the shown effects of finasteride on alcohol cue reactivity may be mediated by alternative mechanisms, such as changes in neurotransmitter systems, stress‐related pathways or neuroinflammation. These findings underline the potential of finasteride as a novel therapeutic approach to problematic alcohol use, emphasizing the need for further longitudinal and mechanistic studies to unravel its exact mechanisms and the multifaceted impacts. Despite the absence of evidence supporting a role of dihydrotestosterone in heavy episodic drinking, our findings nevertheless provide meaningful groundwork for understanding the neurobiological impact of finasteride and suggest that future mechanistic studies should focus on populations with more severe stages of AUD.

## Author Contributions

Conceived and designed the experiments: R.B‐J., I.R., P.B., W.H.S., M.K., S.V‐K., F.K., S.G. and B.L. Performed the experiments: R.B‐J., M.K., M.R., C.M. and B.L. Data preprocessing: R.B‐J., S.H., H.T. and S.G. Analysed the data: R.B‐J., S.H., C.M. and B.L. Wrote the paper: R.B‐J. and B.L. Commented on the manuscript and provided intellectual input: All authors.

## Funding

The project was funded by the Deutsche Forschungsgemeinschaft (DFG, German Research Foundation)—Project‐ID 402170461 – TRR265 [13, 14]. The funders had no role in the study design, data collection, analysis, decision to publish or preparation of the manuscript.

## Conflicts of Interest

The authors declare no conflicts of interest.

## Supporting information


**Figure S1:** Participant flow chart.
**Table S1:** Sample characteristics by sequence.
**Table S2:** Association between dihydrotestosterone concentrations and brain activation in separated models specific to the regions of interest indicated on neutral contrast.
**Table S3:** Association between dihydrotestosterone concentrations, age, time point, medication and sequence with brain activation in models specific to the regions of interest on alcohol contrast.
**Table S4:** Association between dihydrotestosterone concentrations, age, time point, medication and sequence with brain activation in models specific to the regions of interest on neutral contrast.
**Table S5:** Association of dihydrotestosterone concentration with Alcohol Urge Questionnaire and Visual Analogue Scales.
**Table S6:** Association of dihydrotestosterone concentration, time point, medication and sequence with Alcohol Urge Questionnaire and Visual Analogue Scales.
**Table S7:** Medication effects on hormone parameters.

## Data Availability

The data that support the findings of this study are available on request from the corresponding author. The data are not publicly available due to privacy or ethical restrictions.

## References

[1] WHO , Global Status Report on Alcohol and Health, vol. 2018 (World Health Organization, 2018).

[2] B. Lenz , C. Muhle , B. Braun , et al., “Prenatal and Adult Androgen Activities in Alcohol Dependence,” Acta Psychiatrica Scandinavica 136, no. 1 (2017): 96–107. 28383757 10.1111/acps.12725

[3] A. O. Brinkmann , “Molecular Mechanisms of Androgen Action—A Historical Perspective,” Methods in Molecular Biology 776 (2011): 3–24. 21796517 10.1007/978-1-61779-243-4_1

[4] A. Pierucci‐Lagha , J. Covault , R. Feinn , et al., “ *GABRA2* Alleles Moderate the Subjective Effects of Alcohol, Which Are Attenuated by Finasteride,” Neuropsychopharmacology 30, no. 6 (2005): 1193–1203. 15702134 10.1038/sj.npp.1300688

[5] M. M. Ford , N. Yoneyama , M. N. Strong , A. Fretwell , M. Tanchuck , and D. A. Finn , “Inhibition of 5α‐Reduced Steroid Biosynthesis Impedes Acquisition of Ethanol Drinking in Male C57BL/6J Mice,” Alcoholism, Clinical and Experimental Research 32, no. 8 (2008): 1408–1416. 18565155 10.1111/j.1530-0277.2008.00718.x PMC2847609

[6] M. M. Ford , J. D. Nickel , and D. A. Finn , “Treatment With and Withdrawal From Finasteride Alter Ethanol Intake Patterns in Male C57BL/6J Mice: Potential Role of Endogenous Neurosteroids?,” Alcohol 37, no. 1 (2005): 23–33. 16472716 10.1016/j.alcohol.2005.11.002 PMC1533880

[7] J. Covault , T. Pond , R. Feinn , A. J. Arias , C. Oncken , and H. R. Kranzler , “Dutasteride Reduces Alcohol's Sedative Effects in Men in a Human Laboratory Setting and Reduces Drinking in the Natural Environment,” Psychopharmacology 231, no. 17 (2014): 3609–3618. 24557088 10.1007/s00213-014-3487-4 PMC4181572

[8] Y. Mukai , T. Higashi , Y. Nagura , and K. Shimada , “Studies on Neurosteroids XXV. Influence of a 5α‐Reductase Inhibitor, Finasteride, on Rat Brain Neurosteroid Levels and Metabolism,” Biological & Pharmaceutical Bulletin 31, no. 9 (2008): 1646–1650. 18758053 10.1248/bpb.31.1646

[9] G. F. Koob and N. D. Volkow , “Neurobiology of Addiction: A Neurocircuitry Analysis,” Lancet Psychiatry 3, no. 8 (2016): 760–773. 27475769 10.1016/S2215-0366(16)00104-8 PMC6135092

[10] J. Zeng , S. Yu , H. Cao , Y. Su , Z. Dong , and X. Yang , “Neurobiological Correlates of Cue‐Reactivity in Alcohol‐Use Disorders: A Voxel‐Wise Meta‐Analysis of fMRI Studies,” Neuroscience and Biobehavioral Reviews 128 (2021): 294–310. 34171325 10.1016/j.neubiorev.2021.06.031

[11] P. O. Gisleskog , D. Hermann , M. Hammarlund‐Udenaes , and M. O. Karlsson , “A Model for the Turnover of Dihydrotestosterone in the Presence of the Irreversible 5α‐Reductase Inhibitors GI198745 and Finasteride,” Clinical Pharmacology and Therapeutics 64, no. 6 (1998): 636–647. 9871428 10.1016/S0009-9236(98)90054-6

[12] F. Z. Stanczyk , C. G. Azen , and M. C. Pike , “Effect of Finasteride on Serum Levels of Androstenedione, Testosterone and Their 5α‐Reduced Metabolites in Men at Risk for Prostate Cancer,” Journal of Steroid Biochemistry and Molecular Biology 138 (2013): 10–16. 23474436 10.1016/j.jsbmb.2013.02.015

[13] A. Heinz , F. Kiefer , M. N. Smolka , et al., “Addiction Research Consortium: Losing and Regaining Control Over Drug Intake (ReCoDe)—From Trajectories to Mechanisms and Interventions,” Addiction Biology 25, no. 2 (2020): e12866. 31859437 10.1111/adb.12866

[14] R. Spanagel , P. Bach , T. Banaschewski , et al., “The ReCoDe Addiction Research Consortium: Losing and Regaining Control Over Drug Intake—Findings and Future Perspectives,” Addiction Biology 29, no. 7 (2024): e13419. 38949209 10.1111/adb.13419 PMC11215792

[15] S. Vollstädt‐Klein , S. Wichert , J. Rabinstein , et al., “Initial, Habitual and Compulsive Alcohol Use Is Characterized by a Shift of Cue Processing From Ventral to Dorsal Striatum,” Addiction 105, no. 10 (2010): 1741–1749. 20670348 10.1111/j.1360-0443.2010.03022.x

[16] I. Dybek , G. Bischof , J. Grothues , et al., “The Reliability and Validity of the Alcohol Use Disorders Identification Test (AUDIT) in a German General Practice Population Sample,” Journal of Studies on Alcohol 67, no. 3 (2006): 473–481. 16608159 10.15288/jsa.2006.67.473

[17] V. Flaudias , F. Teisseidre , I. De Chazeron , et al., “A Multi‐Dimensional Evaluation of Craving and Impulsivity Among People Admitted for Alcohol‐Related Problems in Emergency Department,” Psychiatry Research 272 (2019): 569–571. 30616125 10.1016/j.psychres.2018.12.118

[18] M. J. Bohn , D. D. Krahn , and B. A. Staehler , “Development and Initial Validation of a Measure of Drinking Urges in Abstinent Alcoholics,” Alcoholism, Clinical and Experimental Research 19, no. 3 (1995): 600–606. 7573780 10.1111/j.1530-0277.1995.tb01554.x

[19] P. Bach , I. Reinhard , A. Koopmann , et al., “Test‐Retest Reliability of Neural Alcohol Cue‐Reactivity: Is There Light at the End of the Magnetic Resonance Imaging Tube?,” Addiction Biology 27, no. 1 (2022): e13069. 34132011 10.1111/adb.13069

[20] C. Rorden and M. Brett , “Stereotaxic Display of Brain Lesions,” Behavioural Neurology 12, no. 4 (2000): 191–200. 11568431 10.1155/2000/421719

[21] R. Zhang , H. Deng , and X. Xiao , “The Insular Cortex: An Interface Between Sensation, Emotion and Cognition,” Neuroscience Bulletin 40, no. 11 (2024): 1763–1773. 38722464 10.1007/s12264-024-01211-4 PMC11607240

[22] T. Saengmearnuparp , B. Lojanapiwat , N. Chattipakorn , and S. Chattipakorn , “The Connection of 5‐Alpha Reductase Inhibitors to the Development of Depression,” Biomedicine & Pharmacotherapy 143 (2021): 112100. 34479019 10.1016/j.biopha.2021.112100

[23] S. C. Godar , R. Cadeddu , G. Floris , et al., “The Steroidogenesis Inhibitor Finasteride Reduces the Response to Both Stressful and Rewarding Stimuli,” Biomolecules 9, no. 11 (2019). 10.3390/biom9110749 PMC6920809 31752360

[24] R. Frau , G. Pillolla , V. Bini , S. Tambaro , P. Devoto , and M. Bortolato , “Inhibition of 5α‐Reductase Attenuates Behavioral Effects of D1‐, but Not D2‐Like Receptor Agonists in C57BL/6 Mice,” Psychoneuroendocrinology 38, no. 4 (2013): 542–551. 22877998 10.1016/j.psyneuen.2012.07.014 PMC3540184

[25] R. Frau , L. J. Mosher , V. Bini , et al., “The Neurosteroidogenic Enzyme 5α‐Reductase Modulates the Role of D1 Dopamine Receptors in Rat Sensorimotor Gating,” Psychoneuroendocrinology 63 (2016): 59–67. 26415119 10.1016/j.psyneuen.2015.09.014 PMC4695380

[26] A. Soggiu , C. Piras , V. Greco , et al., “Exploring the Neural Mechanisms of Finasteride: A Proteomic Analysis in the Nucleus Accumbens,” Psychoneuroendocrinology 74 (2016): 387–396. 27750143 10.1016/j.psyneuen.2016.10.001

[27] J. P. Schacht , R. F. Anton , and H. Myrick , “Functional Neuroimaging Studies of Alcohol Cue Reactivity: A Quantitative Meta‐Analysis and Systematic Review,” Addiction Biology 18, no. 1 (2013): 121–133. 22574861 10.1111/j.1369-1600.2012.00464.x PMC3419322

[28] H. Ekhtiari , M. Zare‐Bidoky , A. Sangchooli , et al., “A Methodological Checklist for fMRI Drug Cue Reactivity Studies: Development and Expert Consensus,” Nature Protocols 17, no. 3 (2022): 567–595. 35121856 10.1038/s41596-021-00649-4 PMC9063851

[29] P. Bach , J. Zaiser , S. Zimmermann , et al., “Stress‐Induced Sensitization of Insula Activation Predicts Alcohol Craving and Alcohol Use in Alcohol Use Disorder,” Biological Psychiatry 95, no. 3 (2024): 245–255. 37678541 10.1016/j.biopsych.2023.08.024

[30] A. Beck , T. Wustenberg , A. Genauck , et al., “Effect of Brain Structure, Brain Function, and Brain Connectivity on Relapse in Alcohol‐Dependent Patients,” Archives of General Psychiatry 69, no. 8 (2012): 842–852. 22868938 10.1001/archgenpsychiatry.2011.2026

[31] S. Kim and D. Lee , “Prefrontal Cortex and Impulsive Decision Making,” Biological Psychiatry 69, no. 12 (2011): 1140–1146. 20728878 10.1016/j.biopsych.2010.07.005 PMC2991430

[32] D. J. Schad , M. Garbusow , E. Friedel , et al., “Neural Correlates of Instrumental Responding in the Context of Alcohol‐Related Cues Index Disorder Severity and Relapse Risk,” European Archives of Psychiatry and Clinical Neuroscience 269, no. 3 (2019): 295–308. 29313106 10.1007/s00406-017-0860-4

[33] J. D. McConnell , J. D. Wilson , F. W. George , J. Geller , F. Pappas , and E. Stoner , “Finasteride, an Inhibitor of 5 Alpha‐Reductase, Suppresses Prostatic Dihydrotestosterone in Men With Benign Prostatic Hyperplasia,” Journal of Clinical Endocrinology and Metabolism 74, no. 3 (1992): 505–508. 1371291 10.1210/jcem.74.3.1371291

