# Supplementary material for: Dihydrotestosterone and Finasteride Effects on Alcohol Cue‐Elicited Brain Activity in Males With Heavy Episodic Drinking
Source: Addict Biol. 2026 Jan 28;31(2):e70123. doi: 10.1111/adb.70123 (PMC12848907; doi:10.1111/adb.70123)
Supplement: Supplementary file 1 — Figure S1: Participant flow chart. Table S1: Sample characteristics by sequence. Table S2: Association between dihydrotestosterone concentrations and brain activation in separated models specific to the regions of interest indicated on neutral contrast. Table S3: Association between dihydrotestosterone concentrations, age, time point, medication and sequence with brain activation in models specific to the regions of interest on alcohol contrast. Table S4: Association between dihydrotestosterone concentrations, age, time point, medication and sequence with brain activation in models specific to the regions of interest on neutral contrast. Table S5: Association of dihydrotestosterone concentration with Alcohol Urge Questionnaire and Visual Analogue Scales. Table S6: Association of dihydrotestosterone concentration, time point, medication and sequence with Alcohol Urge Questionnaire and Visual Analogue Scales. Table S7: Medication effects on hormone parameters. [file ADB-31-e70123-s001.pdf]

Supplemental Material

Supplementary Figure 1

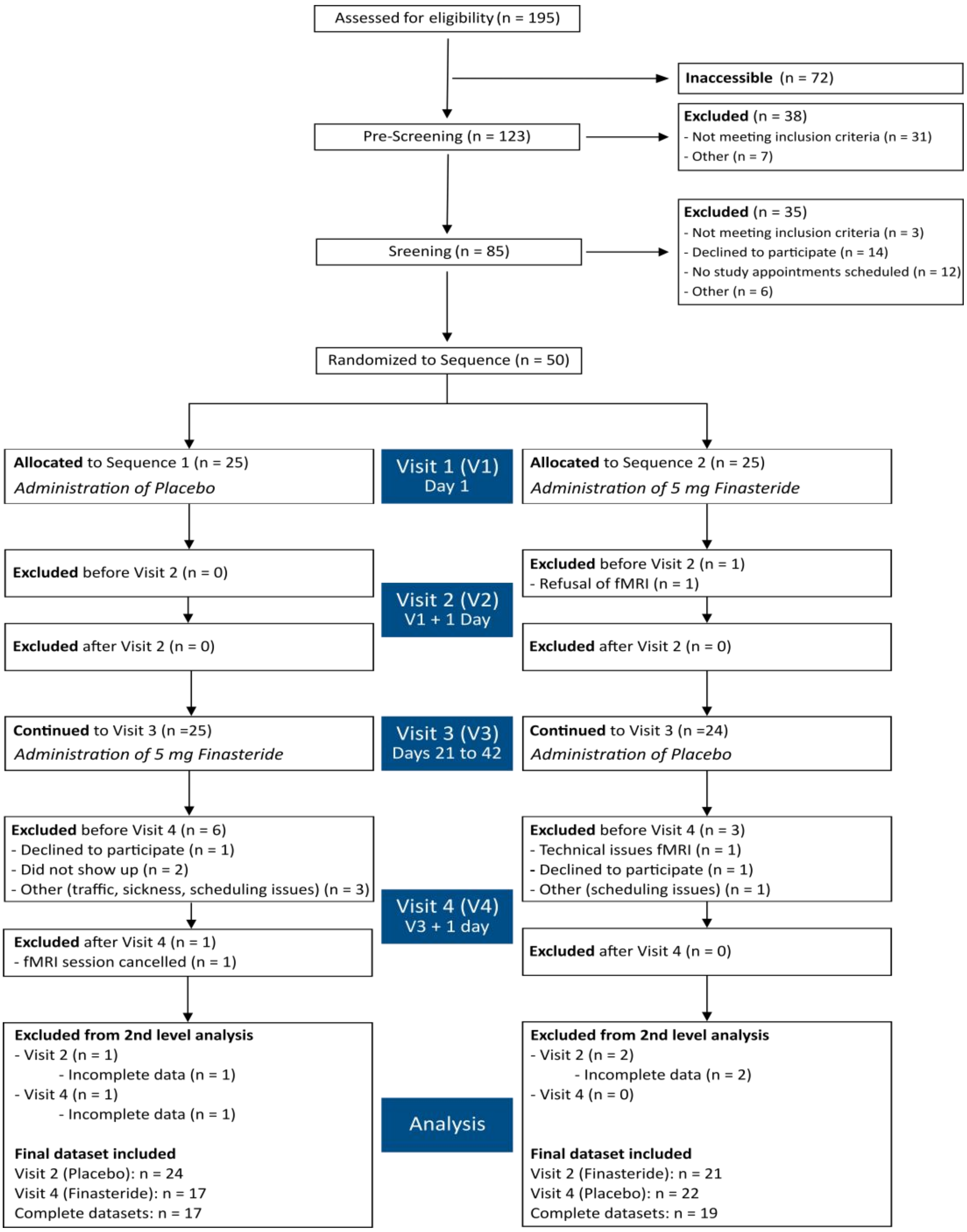

Caption Supplementary Figure 1. Participant flow chart

Supplementary Table 1. Sample characteristics by sequence

Sequence	Finasteride – Placebo			Placebo – Finasteride			
	N	M	SD	N	M	SD	
Age	25	35.92	14.98	25	33.04	13.03	1.61, t(50) = 0.55, p = 0.581
AUDIT	24	10.52	4.97	24	11.83	4.97	-0.83, t(48) = -0.80, p = 0.423
Height (cm)	25	181.36	8.04	25	182.72	7.14	-1.76, t(50) = -1.15, p = 0.254
Weight (kg)	25	82.92	13.35	25	81.8	9.75	0.64, t(50) = 0.27, p = 0.790
Body mass index (kg/m²)	25	25.09	3.49	25	24.06	1.88	1.41, t(50) = 0.53, p = 0.515

Sample characteristics by sequence and statistics for mean differences (independent samples t tests). AUDIT, Alcohol Use Disorders Identification Test; HDD, heavy drinking days; M, mean; N, number of participants; SD, standard deviation.

Supplementary Table 2. Association between dihydrotestosterone concentrations and brain activation in separated models specific to the regions of interest indicated on neutral contrast

	β	95% CI	F	df1; df2	p
N[cases] = 48					
N[observations] = 84					
**Right caudate**					
Dihydrotestosterone concentrations	-0.001	[-0.001; 0.001]	1;141	1; 38	0.650
Age	-0.001	[-0.004; 0.001]	1.277	1; 77	0.262
Smoking status	-0.014	[-0.087; 0.059]	0.137	1; 77	0.712
**Left caudate**					
Dihydrotestosterone concentrations	0.000	[-0.001; -0.001]	2.836	1; 77	0.962
Age	-0.002	[-0.005; 0.002]	1.215	1; 24	0.281
Smoking status	-0.011	[-0.100; 0.078]	0.059	1; 25	0.809
**Left superior frontal gyrus orbital**					
Dihydrotestosterone concentrations	0.000	[-0.001; 0.000]	0.836	1; 46	0.365
Age	-0.003	[-0.009; 0.003]	1.408	1; 25	0.316
Smoking status	0.080	[-0.079; 0.240]	1.070	1; 26	0.311
**Right superior frontal gyrus**					
Dihydrotestosterone concentrations	0.000	[-0.001; -0.000]	0.733	1; 53	0.396
Age	-0.003	[-0.008; 0.003]	0.882	1; 19	0.359
Smoking status	-0.006	[-0.158; 0.147]	0.006	1; 19	0.940
**Right middle frontal gyrus**					
Dihydrotestosterone concentrations	-0.001	[-0.001; 0.000]	1.497	1; 77	0.225
Age	-0.002	[-0.005; 0.001]	1.790	1; 77	0.185
Smoking status	-0.012	[-0.102; 0.079]	0.066	1; 77	0.798
**Right precentral gyrus**					
Dihydrotestosterone concentrations	0.000	[0.000; 0.001]	1.392	1; 53	0.243
Age	0.001	[-0.004; 0.006]	0.214	1; 37	0.647
Smoking status	-0.043	[-0.181; 0.094]	0.405	1; 39	0.528
**Right insula**					
Dihydrotestosterone concentrations	-0.001	[-0.001; 0.001]	5.882	1; 49	0.183
Age	-0.002	[-0.007; 0.002]	1.941	1; 5	0.225
Smoking status	-0-005	[-0.123; 0.113]	0.012	1; 5	0.916
**Left insula**					
Dihydrotestosterone concentrations	-0.001	[-0.001; 0.001]	1.141	1; 38	0.650
Age	-0.002	[-0.005; 0.002]	1.141	1; 26	0.295
Smoking status	0.022	[-0.075; 0.119]	0.211	1; 28	0.650

The multilevel models include fixed and random intercepts. 95% CI, 95% confidence interval; β, regression coefficient.

Supplementary Table 3. Association between dihydrotestosterone concentrations, age, time point , medication, and sequence with brain activation in models specific to the regions of interest on alcohol contrast

	β	95% CI	F	df1; df2	p
N[cases] = 48					
N[observations] = 84					
**Right caudate**					
Dihydrotestosterone concentrations	0.000	[-0.001; 0.001]	0.002	1; 53	0.962
Age	-0.004	[-0.013; 0.005]	0.667	1; 46	0.418
Smoking status	-0.163	[-0.402; 0.076]	1.886	1; 43	0.177
Time point (V2 versus V4)			0.630	1; 45	0.432
V2	0.077	[-0.118; 0.272]			

V4	[Reference]				
Medication			5.923	1; 50	**0.019**
Finasteride	0.246	[0.043; 0.449]			
Placebo	[Reference]				
Sequence			0.238	1; 44	0.628
Placebo-finasteride	0.054	[-0.170; 0.279]			
Finasteride-placebo	[Reference]				
**Left caudate**					
Dihydrotestosterone concentrations	0.000	[-0.001; 0.001]	0.048	1; 51	0.828
Age	-0.007	[-0.015; 0.000]	3.727	1; 44	0.060
Smoking status	-0.170	[-0.369; 0.029]	2.982	1; 41	0.092
Time point (V2 versus V4)			0.002	1; 43	0.969
V2	-0.003	[-0.166; 0.159]			
V4	[Reference]				
Medication			4.027	1; 48	**0.049**
Finasteride	0.170	[0.001; 0.339]			
Placebo	[Reference]				
Sequence			0.078	1; 41	0.782
Placebo-finasteride	0.026	[-0.161; 0.213]			
Finasteride-placebo	[Reference]				
**Left superior frontal gyrus orbital**					
Dihydrotestosterone concentrations	0.000	[-0.002; 0.002]	0.000	1; 58	0.994
Age	-0.003	[-0.018; 0.011]	0.214	1; 47	0.646
Time point (V2 versus V4)			0.292	1; 43	0.592
V2	0.071	[-0.195; 0.338]			
V4	[Reference]				
Medication			1.557	1; 49	0.218
Finasteride	0.174	[-0.106; 0.454]			
Placebo	[Reference]				
Sequence			0.061	1; 58	0.807
Placebo-finasteride	0.174	[-0.106; 0.454]			
Finasteride-placebo	[Reference]				
**Right superior frontal gyrus**					
Dihydrotestosterone concentrations	-0.000	[-0.002; 0.001]	0.554	1; 60	0.460
Age	-0.001	[-0.014; 0.011]	0.048	1; 48	0.828
Smoking status	-0.187	[-0.589; 0.082]	2.311	1; 45	0.135
Time point (V2 versus V4)			0.291	1; 43	0.593
V2	-0.058	[-0.274; 0.158]			
V4	[Reference]				
Medication			4.945	1; 49	**0.031**
Finasteride	0.253	[0.024; 0.481]			
Placebo	[Reference]				
Sequence			1.435	1; 45	0.237
Placebo-Finasteride	-0.187	[-0.502; 0.128]			
Finasteride-Placebo	[Reference]				
**Right middle frontal gyrus**					
Dihydrotestosterone concentrations	-0.002	[-0.002; 0.001]	0.701	1; 58	0.406
Age	-0.002	[-0.014; 0.010]	0.120	1; 46	0.731
Smoking status	-0.212	[-0.533; 0.109]	1.768	1; 43	0.191
Time point (V2 versus V4)			0.172	1; 42	0.681
V2	-0.044	[-0.258; 0.170]			
V4	[Reference]				
Medication			2.114	1; 48	0.152
Finasteride	0.163	[-0.062; 0.388]			
Placebo	[Reference]				
Sequence			0.261	1; 48	0.612
Placebo-finasteride	-0.076	[-0.378; 0.225]			
Finasteride-placebo	[Reference]				
**Right pre central gyrus**					
Dihydrotestosterone concentrations	0.002	[-0.000; 0.004]	2.946	1; 59	0.091
Age	0.002	[-0.012; 0.017]	0.093	1; 46	0.762
Smoking status	-0.179	[-0.563; 0.205]	0.883	1; 43	0.353
Time point (V2 versus V4)			0.021	1; 41	0.913
V2	0.014	[-0.236; 0.263]			

	β	95% CI	F	df1; df2	p
V4	[Reference]				
Medication			3.915	1; 47	0.054
Finasteride	0.259	[-0.004; 0.522]			
Placebo	[Reference]				
Sequence			0.677	1; 43	0.415
Placebo-finasteride	-0.147	[-0.508; 0.214]			
Finasteride-placebo	[Reference]				
**Right insula**					
Dihydrotestosterone concentrations	-0.001	[-0.002; 0.001]	0.532	1; 56	0.496
Age	-0.001	[-0.011; 0.009]	0.024	1; 45	0.878
Smoking status	-0.356	[-0.616; -0.096]	7.650	1; 42	0.880
Time point (V2 versus V4)			0.027	1; 42	0.871
V2	-0.015	[-0.197; 0.167]			
V4	[Reference]				
Medication			3.528	1; 47	0.066
Finasteride	0.178	[-0.013; 0.369]			
Placebo	[Reference]				
Sequence			0.663	1; 43	0.420
Placebo-finasteride	-0.099	[-0.343; 0.146]			
Finasteride-placebo	[Reference]				
**Left insula**					
Dihydrotestosterone concentrations	0.000	[-0.001; 0.002]	0.104	1; 52	0.749
Age	-0.005	[-0.015; 0.004]	1.383	1; 45	0.246
Smoking status	-0.213	[-0.461; 0.035]	3.005	1, 42	0.090
Time point (V2 versus V4)			0.118	1; 44	0.733
V2	0.034	[-0.164; 0.232]			
V4	[Reference]				
Medication			4.169	1; 49	**0.047**
Finasteride	0.210	[0.003; 0.416]			
Placebo	[Reference]				
Sequence			0.013	1; 43	0.909
Placebo-finasteride	0.013	[-0.220; 0.247]			
Finasteride-placebo	[Reference]				

The multilevel models include fixed and random intercepts. 95% CI, 95% confidence interval; β, regression coefficient. P < .05 in bold.

Supplementary Table 4. Association between dihydrotestosterone concentrations, age, time point , medication, and sequence with brain activation in models specific to the regions of interest on neutral contrast

	β	95% CI	F	df1; df2	p
N[cases] = 48					
N[observations] = 84					
**Right caudate**					
Dihydrotestosterone concentrations	-0.000	[-0.001; 0.002]	3.433	1; 74	0.068
Age	-0.002	[-0.004; 0.001]	1.302	1; 74	0.257
Smoking status	-0.010	[-0.084; 0.063]	0.076	1; 74	0.784
Time point (V2 versus V4)			0.037	1; 74	0.848
V2	-0.006	[-0.073; 0.061]			
V4	[Reference]				
Medication			0.148	1; 74	0.701
Finasteride	0.013	[-0.005; 0.081]			
Placebo	[Reference]				
Sequence			2.997	1; 74	0.088
Placebo-finasteride	0.059	[-0.009; 0.127]			
Finasteride-placebo	[Reference]				
**Left caudate**					
Dihydrotestosterone concentrations	-0.000	[-0.001; 0.007]	3.097	1; 37	0.087
Age	-0.002	[-0.005; 0.002]	1.124	1; 23	0.300
Smoking status	-0.005	[-0.095; 0.085]	0.013	1; 23	0.909
Time point (V2 versus V4)			0.447	1; 24	0.510
V2	0.022	[-0.046; 0.091]			
V4	[Reference]				
Medication			0.167	1; 27	0.686
Finasteride	0.014	[-0.056; 0.084]			

Placebo	[Reference]				
Sequence			1.824	1; 23	0.190
Placebo-finasteride	0.054	[-0.029; 0.137]			
Finasteride-placebo	[Reference]				
**Left superior frontal gyrus orbital**					
Dihydrotestosterone concentrations	-0.001	[-0.001; 0.000]	1.387	1; 41	0.246
Age	-0.003	[-0.009; 0.003]	1.044	1; 23	0.318
Smoking status	0.093	[-0.074; 0.260]	1.328	1; 24	0.261
Time point (V2 versus V4)			0.309	1; 23	0.584
V2	0.031	[-0.086; 0.149]			
V4	[Reference]				
Medication			0.998	1; 27	0.327
Finasteride	-0.058	[-0.178; 0.062]			
Placebo	[Reference]				
Sequence			0.189	1; 23	0.667
Placebo-finasteride	0.023	[-0.122; 0.187]			
Finasteride-placebo	[Reference]				
**Right superior frontal gyrus**					
Dihydrotestosterone concentrations	-0.000	[-0.001; 0.000]	1.326	1; 74	0.253
Age	-0.002	[-0.005; 0.001]	1.705	1; 75	0.196
Smoking status	-0.002	[-0.162; 0.158]	0.054	1; 74	0.984
Time point (V2 versus V4)			0.065	1; 76	0.800
V2	0.011	[-0.073; 0.095]			
V4	[Reference]				
Medication			0.750	1; 75	0.389
Finasteride	0.037	[-0.049; 0.123]			
Placebo	[Reference]				
Sequence			0.632	1; 74	0.429
Placebo-finasteride	0.034	[-0.051; 0.119]			
Finasteride-placebo	[Reference]				
**Right middle frontal gyrus**					
Dihydrotestosterone concentrations	0.000	[-0.001; 0.001]	0.5273	1; 74	0.253
Age	-0.002	[-0.008; 0.003]	0.691	1; 72	0.196
Smoking status	-0.012	[-0.104; 0.081]	0.069	1; 74	0.800
Time point (V2 versus V4)			0.574	1; 74	0.458
V2	0.033	[-0.058; 0.123]			
V4	[Reference]				
Medication			0.902	1; 74	0.352
Finasteride	0.043	[-0.051; 0.137]			
Placebo	[Reference]				
Sequence			0.869	1; 73	0.362
Placebo-finasteride	0.066	[-0.082; 0.214]			
Finasteride-placebo	[Reference]				
**Right pre central gyrus**					
Dihydrotestosterone concentrations	0.000	[0.000; 0.001]	1.303	1; 50	0.259
Age	0.001	[-0.004; 0.006]	0.218	1; 36	0.643
Smoking status	-0.045	[-0.186; 0.097]	0.411	1; 38	0.525
Time point (V2 versus V4)			0.032	1; 39	0.858
V2	-0.010	[-0.121; 0.102]			
V4	[Reference]				
Medication			1.076	1; 42	0.306
Finasteride	0.059	[-0.055; 0.173]			
Placebo	[Reference]				
Sequence			1.172	1; 37	0.286
Placebo-finasteride	0.070	[-0.061; 0.200]			
Finasteride-placebo	[Reference]				
**Right insula**					
Dihydrotestosterone concentrations	-0.001	[-0.001; -0.000]	5.021	1; 76	0.060
Age	-0.002	[-0.007; 0.003]	1;811	1; 75	0.252
Smoking status	-0.005	[-0.139; 0.129]	0.012	1; 75	0.918
Time point (V2 versus V4)			0.003	1; 76	0.959
V2	-0.002	[-0.115; 0.110]			
V4	[Reference]				
Medication			0.597	1; 75	0.472

Finasteride	0.034	[-0.076; 0.144]			
Placebo	[Reference]				
Sequence			0.874	1; 74	0.401
Placebo-finasteride	0.042	[-0.082; 0.167]			
Finasteride-placebo	[Reference]				
**Left insula**					
Dihydrotestosterone concentrations	-0.001	[-0.001; -0.005]	5.275	1; 74	0.062
Age	-0.002	[-0.005; 0.002]	1.116	1; 74	0.249
Smoking status	0.024	[-0.071; 0.119]	0.256	1; 74	0.614
Time point (V2 versus V4)			0.039	1; 74	0.844
V2	0.009	[-0.078; 0.095]			
V4	[Reference]				
Medication			0.289	1; 74	0.592
Finasteride	0.024	[-0.064; 0.112]			
Placebo	[Reference]				
Sequence			1.095	1; 74	0.299
Placebo-finasteride	0.046	[-0.042; 0.134]			
Finasteride-placebo	[Reference]				

The multilevel models include fixed and random intercepts. 95% CI, 95% confidence interval; β, regression coefficient. P < .05 in bold.

Supplementary Table 5. Association of dihydrotestosterone concentration with Alcohol Urge Questionnaire and Visual Analogue Scales

	β	95% CI	F	df1; df2	p
N[cases] = 82					
N[observations] = 282					
**AUQ**					
Dihydrotestosterone concentrations	-0.001	[-0.001; 0.005]	0.027	1; 143	0.869
Age	-0.005	[-0.015; 0.005]	1.122	1; 46	0.295
Smoking status	-0.160	[0.245; -0.438]	1.386	1; 45	0.245
**VAS 1 (How strong is your craving for alcohol now?)**					
Dihydrotestosterone concentrations	-0.030	[-0.069; 0.009]	2.356	1; 49	0.126
Age	-0.526	[-0.933; -0.118]	6.732	1; 49	0.112
Smoking status	-3.763	[-15.181; 7.655]	0.440	1;46	0.510
**VAS 2 (How strong is your intent to drink alcohol now?)**					
Dihydrotestosterone concentrations	-0.043	[-0.077; -0.008-]	6.009	1; 221	0.115
Age	-0.317	[-0.692; -0.058]	2.882	1; 49	0.096
Smoking status	-1.685	[-12.216; 8.845]	0.104	1; 46	0.749
**VAS 3 (Would you expect a positive effect if you took alcohol now?)**					
Dihydrotestosterone concentrations	-0.018	[-0.055; 0.019]	0.947	1; 246	0.331
Age	-0.621	[-1.067; -0.176]	7.849	1; 49	0.070
Smoking status	1.087	[-11.462; 13.637]	0.030	1; 47	0.862
**VAS 4 (Would you expect a negative condition to be improved or physical withdrawal symptoms to be alleviated by taking alcohol?)**					
Dihydrotestosterone concentrations	0.001	[-0.031; 0.032]	0.001	1; 230	0.937
Age	-0.034	[-0.396; 0.327]	0.036	1; 47	0.850
Smoking status	-5.984	[-16.150; 4.182]	1.405	1; 45	0.242
**VAS 5 (How strong is your wish to not drink alcohol?)**					
Dihydrotestosterone concentrations	0.020	[-0.043; 0.083]	0.391	1; 131	0.533
Age	-0.697	[0.303; 1.418]	9.654	1; 46	0.065
Smoking status	14.551	[-0.967; 30.069]	3.570	1; 44	0.383

The multilevel models include fixed and random intercepts. 95% CI, 95% confidence interval; β, regression coefficient. P < .05 in bold.

Supplementary Table 6. Association of dihydrotestosterone concentration, time point, medication, and sequence with Alcohol Urge Questionnaire and Visual Analogue Scales

	β	95% CI	F	df1; df2	p
N[cases] = 82					
N[observations] = 282					
**AUQ**					
Dihydrotestosterone concentrations	-6.001	[-0.001; 0.001]	0.011	1; 123	0.916
Age	-0.005	[-0.015; -0.005]	1.085	1; 45	0.303
Smoking status	-0.181	[-0.461; 0.099]	1.703	1; 44	0.199
Within study visit time point			4.380	1; 216	**0.014**
Baseline	-0.166	[-0.290; -0.042]			
Post MRI	-0.010	[-0.134; 0.114]			
Post BarLab	[Reference]				
Time point (V2 versus V4)			4.299	1; 226	**0.039**
V2	0.111	[0.006; 0.217]			
V4	[Reference]				
Medication			0.001	1; 249	0.981
Finasteride	-0.001	[-0.115; 0.112]			
Placebo	[Reference]				
Sequence			1.393	1; 43	0.244
Placebo-finasteride	-0.150	[-0.406; 0.106]			
Finasteride-placebo	[Reference]				
**VAS 1 (How strong is your craving for alcohol now?)**					
Dihydrotestosterone concentrations	-0.034	[-0.074; 0.007]	2.690	1; 202	0.103
Age	-0.523	[-0.936; -0.110]	1.165	1; 201	**0.014**
Smoking status	-4.145	[-15.937; 7.648]	0.501	1; 45	0.483
Within study visit time point			7.262	1; 220	**0.008**
Baseline	-11.521	[-15.176; -7.866]			
Post MRI	-7.276	[-10.946; -3.605]			
Post BarLab	[Reference]				
Time point (V2 versus V4)			7.262	1; 221	**0.008**
V2	4.286	[1.152; 7.420]			
V4	[Reference]				
Medication			0.125	1; 241	0.727
Finasteride	-0.614	[-4.037; 2.809]			
Placebo	[Reference]				
Sequence			1.657	1; 45	422
Placebo-finasteride	-4.357	[-15.183; 6.469]			
Finasteride-placebo	[Reference]				
**VAS 2 (How strong is your intent to drink alcohol now?)**					
Dihydrotestosterone concentrations	-0.033	[-0.069; 0.003]	3.205	1; 213	0.075
Age	-0.291	[-0.671; 0.090]	2.360	1; 47	0.131
Smoking status	-2.468	[-13.358; 8.422]	0.208	1; 45	0.650
Within study visit time point			16.117	2; 216	0.056
Baseline	-9.462	[-12.665; -6.260]			
Post MRI	-4.683	[-7.899; -1.467]			
Post BarLab	[Reference]				
Time point (V2 versus V4)			3.802	1; 225	0.068
V2	2.739	[-0.065; 5.135]			
V4	[Reference]				
Medication			1.926	1; 241	0.166
Finasteride	2.120	[-0.890; 5.130]			
Placebo	[Reference]				
Sequence			0.315	1; 45	0.577
Placebo-finasteride	-2.788	[-12.786; 7.210]			
Finasteride-placebo	[Reference]				
**VAS 3 (Would you expect a positive effect if you took alcohol now?)**					
Dihydrotestosterone concentrations	-0.003	[-0.032; 0.039]	0.032	1; 225	0.336
Age	-0.021	[-0.389; 0.346]	0.013	1; 48	0.908
Smoking status	1.568	[-11.428; 14.564]	0.059	1; 46	0.809

Within study visit time point			1.434	1; 218	0.232
Baseline	1.655	[-1.068; 4.377]			
Post MRI	-0.690	[-2.295; 2.916]			
Post BarLab	[Reference]				
Time point (V2 versus V4)			1.434	1; 218	0.232
V2	1.655	[-1.068; 4.377]			
V4	[Reference]				
Medication			0.318	1; 240	0.573
Finasteride	0.853	[-2.125; 3.830]			
Placebo	[Reference]				
Sequence			0.435	1; 48	0.513
Placebo-finasteride	3.154	[-6.492; 12.801]			
Finasteride-placebo					
**VAS 4 (Would you expect a negative condition to be improved or physical withdrawal symptoms to be alleviated by taking alcohol?)**					
Dihydrotestosterone concentrations	0.042	[-0.025; 0.109]	1.536	1; 121	0.218
Age	0.901	[0.331; 1.471]	10.180	1; 120	0.103
Smoking status	-5.429	[-15.936; 5.078]	1.085	1; 43	0.303
Within study visit time point			0.019	1; 221	0.913
Baseline	18.709	[11.675; 25.693]			
Post MRI	9.287	[2.150; 14.424]			
Post BarLab	[Reference]				
Time point (V2 versus V4)			1.357	1; 212	0.830
V2	-0.421	[-6.483; 5.642]			
V4	[Reference]				
Medication			0.318	1; 204	0.573
Finasteride	0.853	[-2.124; 3.830]			
Placebo	[Reference]				
Sequence			0.435	1; 43	0.513
Placebo-finasteride	3.154	[-6.491; 12.801]			
Finasteride-placebo	[Reference]				
**VAS 5 (How strong is your wish to not drink alcohol?)**					
Dihydrotestosterone concentrations	0.042	[-0.025; 0.109]	1.536	1; 120	0.218
Age	0.536	[0.391; 1.404]	10.201	1; 43	0.003
Smoking status	15.132	[-1.022; 31.286]	3.578	1; 41	0.066
Within study visit time point			13.578	2; 212	**0.000**
Baseline	18.779	[11.675; 25.883]			
Post MRI	9.287	[2.150; 16.424]			
Post BarLab	[Reference]				
Time point (V2 versus V4)			0.019	1; 221	0.891
V2	-0.421	[-6.483; 5.642]			
V4	[Reference]				
Medication			4.086	1; 245	**0.044**
Finasteride	6.652	[0.170; 13.134]			
Placebo	[Reference]				
Sequence			1.928	1; 41	0.173
Placebo-finasteride	10.189	[-4.633; 25.011]			
Finasteride-placebo	[Reference]				

The multilevel models include fixed and random intercepts. 95% CI, 95% confidence interval; β, regression coefficient. P < .05 in bold.

Supplementary Table 7. Medication effects on hormone parameters

	β	95% CI	F	df1; df2	p
N[cases] = 84					
N[observations] = 184					
**Dihydrotestosterone (pg/mL)**					
Medication			8.566	1; 131	**0.004**
Finasteride	-31.977	[14.081; 49.874]			
Placebo	[Reference]				
Age	-2.052	[-3.751; -0.353]	5.885	1; 47	**0.019**
Smoking status					
Blood from V1/V3 versus V2/V4			4.766	1; 133	**0.031**
Study visit 2/4	28.557	[9.873; 47.241]			
Study visit 1/3	[Reference]				
Medication x Blood from V1/V3 versus V2/V4	-26.139	[50.809; 1.470]	4.395	1; 129	**0.038**
Time point			0.615	1; 131	0.434
Study visit 3/4	5.089	[-7.752; 17.930]			
Study visit 1/2	[Reference]				
Sequence	-23.2021	[-70.336; 23.934]	0.980	1; 47	0.327
Time of blood collection	-3.837	[-6.963; -0.711]	5.885	1; 145	**0.016**
**Testosterone (ng/mL)**					
Medication			0.114	1; 132	0.736
Finasteride	0.156	[-0.444; 0.132]			
Placebo	[Reference]				
Age	-0.027	[-0.052;-0.003]	4.946	1; 47	0.331
Smoking status					
Blood from V1/V3 versus V2/V4			5.803	1; 134	**0.017**
Study visit 2/4	-0.395	[-0.696; 0.095]			
Study visit 1/3	[Reference]				
Medication x Blood from V1/V3 versus V2/V4	0.241	[-0.156; 0.638]	1.425	1; 129	0.232
Time point			0.237	1; 132	0.627
Study visit 3/4	-0.051	[-0.257; 0.156]	0.178		
Study visit 1/2	[Reference]				
Sequence	-0.602	[-0.052; -0.003]	3.127	1; 47	0.083
Time of blood collection	-0.083	[-0.133; -0.033]	10.262	1; 48	**0.001**
**Allopregnanolone (pg/mL)**					
Medication			6.032	1; 129	0.115
Finasteride	6.514	[-12.475; 10.403]			
Placebo	[Reference]				
Age	-0.399	[-1.112; 0.314]	1.270	1; 46	0.266
Smoking status					
Blood from V1/V3 versus V2/V4			0.849	1; 131	0.359
Study visit 2/4	1.527	[-4.780; 7.834]			
Study visit 1/3	[Reference]				
Medication x Blood from V1/V3 versus V2/V4	1.364	[-6.953; 9.681]	0.105	1; 128	0.746
Time point			0.037	1; 130	0.848
Study visit 3/4	0.420	[-3.916; 4.756]			
Study visit 1/2	[Reference]				
Sequence	-1.250	[-32.254; 7.250]	1.623	1; 46	0.209
Time of blood collection	-0.142	[-1.207; 0.923]	0.070	1; 139	0.792

The multilevel models include fixed and random intercepts. 95% CI, 95% confidence interval; β, regression coefficient. P < .05 in bold.
